# COVID-19-Related Long-Term Taste Impairment: Symptom Length, Related Taste, Smell Disturbances, and Sample Characteristics

**DOI:** 10.7759/cureus.38055

**Published:** 2023-04-24

**Authors:** Pedro Cardoso Soares, Patrícia Moreira de Freitas, Carlos de Paula Eduardo, Luciane Hiramatsu Azevedo

**Affiliations:** 1 Special Laboratory of Lasers in Dentistry, Department of Operative Dentistry, School of Dentistry, Universidade de São Paulo, São Paulo, BRA

**Keywords:** loss of taste, taste disturbance, bitter taste receptors (t2rs), taste and smell dysfunctions, covid 19

## Abstract

Introduction: The COVID-19 infection triggered in some patients a prolonged reduction in the perception of both gustatory and olfactory senses (ageusia and anosmia). These symptoms could be manifested during the first days after the contagion, acting as predictors of COVID-19 infection, and additionally, they could be the only symptoms manifested at all. Clinical resolution of anosmia and ageusia was expected to occur within a few weeks, yet in some cases, patients began to demonstrate COVID-19-related long-term taste impairment (CRLTTI), a condition that can persist for longer than two months, contradicting initial evidence.

Objectives: The authors’ aimed to describe the characteristics of the sample of 31 participants with COVID-19-related long-term taste impairment, and their capacity to quantify taste and rate their smell perception.

Material and Methods: Participants were submitted to a taste evaluation of four hyper-concentrated tastes perceived by the tongue (0-10), self-reported their smell (0-10), and answered a semi-structured questionnaire.

Results: Different tastes seemed to be affected differently by COVID-19, despite the lack of statistical relevance observed in this study. Dysgeusia was only expressed in bitter, sweet, and acidic tastes. The mean age observed was 40.2 (SD 12.06) years, with women representing 71% of the sample. Taste impairment persisted for an average period of 10.8 months (SD 5.7). Self-reported smell impairment was reported by the majority of participants with taste impairment. Non-vaccinated people represented 80.6% of the sample.

Conclusions: COVID-19 infection could trigger taste and smell disturbances that lasted as long as 24 months. CRLTTI seems not to affect the four main taste perceptions (hyper-concentrated) equally. Women represented the majority of the sample, with an average age of 40 years (SD 12.06). Previous diseases, medication use, and behavioral aspects seem not to be linked to CRLTTI development.

## Introduction

Throughout the last two years, the COVID-19 outbreak imposed a challenge on the global population, leading to the collapse of health systems and imposing direct changes on the daily lives of everyone. It was also becoming a baptism by fire of 21st-century science that expanded the boundaries of knowledge in search of fast and suitable solutions to combat COVID-19.

Since the early reports, some of the most prevalent symptoms associated with COVID-19 infection have been the perception of a reduction in and/or gustatory and olfactory distortions. Furthermore, in some cases, these were the first and only symptoms manifested during the infectious phase (IP) [1]. The clinical resolution of anosmia and ageusia was expected to occur within a few weeks, yet in some cases, patients began to demonstrate COVID-19-related long-term taste impairment (CRLTTI), a condition that can persist for longer than two months, contradicting initial evidence [2,3].

The lack of knowledge about COVID-19's late manifestations and sequelae has left many questions without clear answers, with some patients reporting prolonged manifestations of symptoms after COVID-19 contagion that persisted for months or even years. The absence or distortion of taste perception directly impacts the quality of life [4-6].

In our study, we assessed the perception of hyper-concentrated substances with the use of four substances perceived by the tongue: self-reported smell, the length of time of taste impairment, and medical history that could predict CRLTTI.

## Materials and methods

Thirty-one patients who participated in a randomized clinical trial (RCT) conducted specifically to treat CRLTTI at the Special Laboratory of Lasers in Dentistry (LELO - Laboratório Especial de Lasers em Odontologia) at the School of Dentistry, University of São Paulo, Brazil, answered a questionnaire (Appendix) and were assessed for taste impairment in the first session of the RCT. This RCT was approved by the local Ethics Committee (CAAE: 46075021.2.0000.0075, Protocol number 4.971.869), registered in the Brazilian Clinical Trials Database (ReBEC - Protocol RBR-3tpvp8t), and also registered with the Universal Trial Number (UTN - U1111-1281-7082). This convenience sample was obtained by posts on social media (non-promoted and exclusively through the official pages of the University of São Paulo Faculty of Dentistry profile on Instagram and Facebook) and divulged via regular media (report on TV, open channel, City of São Paulo - SP). This study refers to the first visit of participants who were included in this RCT. 

To be considered for inclusion in the study of CRLTTI, patients had to have self-reported taste impairment for a period longer than two months, with proven PCR or antigen tests dating back to a minimum of two months before their inclusion in this study. Both vaccinated (full or partial scheme) and non-vaccinated participants were considered for study inclusion. 

All participants were older than 18 years at the time of inclusion in the study. Pregnancy, history of head and neck radiotherapy and/or active oncologic treatment, any condition that led to taste impairment before COVID-19 contagion, chronic medication use capable of causing taste impairment, or xerostomia were all considered exclusion criteria. 

The questionnaire included binary questions focusing on previous medical conditions, continuous medication use, sex, medication hypersensitivity, the need for hospitalization during COVID-19 IP, olfactory loss, persistent symptoms post-IP, and medications used during IP: chloroquine, azithromycin, ivermectin, and corticosteroids. Study participants were also questioned about quantitative outcomes, such as age and length of taste impairment. 

Participants evaluated four hyper-concentrated tastes perceived by the tongue, namely sweet, salty, sour, and bitter. They were blindfolded and received 5 ml of hyper-concentrated solutions, and after 3 seconds, they were asked to identify (open question) the taste and quantify it in a 0-10 range of substance strength, with 0 being complete taste absence and 10 being very strong. Participants were not briefed about which substances they would receive. The sum of all tastes (0-10 for each, sum of all totals of 40) was considered for statistical analysis (0-40 scale).

Taste distortion was considered at the same time as regular evaluation (salt, acid, bitter, and sweet), and it was characterized by the perception of unpleasant taste and/or non-food-related taste perception during the same exposition of hyper-concentrated solutions. If reporting taste distortion, participants were asked to quantify the distortion from 0-10 for each substance. For evaluation analysis, the same concept of the taste perception test was used: 0-10 for each substance and the sum of all (0-40 scale). For statistical purposes, we converted taste distortion results into binomials (yes and no), with zero representing the absence of taste distortion and one and above converted into taste distortion for the evaluated substance (sweet, acidic, bitter, or salt).

In cases where both taste and dysgeusia were described, both scores were registered, for example (coffee taste = 8; chemical substance = 5 when exposed to a bitter solution). This taste evaluation is an adaptation of the Kale method [7]. 

All necessary terms of free and informed consent were obtained before beginning with the clinical tests.

All quantitative and qualitative data were analyzed using Jamovi (The Jamovi project (2021). Jamovi (Version 1.6) [Computer Software]. Retrieved from https://www.jamovi.org). Both qualitative and quantitative outcomes were described by means of descriptive statistics. Quantitative outcomes were described using both the median and mean, despite the non-parametric data. Qualitative outcomes were described in frequency (n and %). Linear regression models were carried out using as dependent variables taste perception (0-40), taste distortion (0-40) and smell perception (0-10). 

## Results

The total sample size was 31 participants. The mean age was 40.26 years (SD 12.06), with women representing 71% (n=22) of the sample, compared with 29% of men (n=9). Tables 1, 2 depict other characteristics of the CRLTTI population analyzed and medication used during the infectious phase, respectively.

**Table 1:** Characteristics of the sample (n=31) with COVID-19-related long-term taste impairment *Medication refers to any drug/vitamin use, not necessarily related to the existent pathological condition.

Chronic Diseases	Medication* use (before COVID19)	Presence of other "long COVID-19" symptoms	Hypersensitivity (food/drug)	Active smokers	Hospitalization due to COVID-19
9 (29%)	18 (58.1%)	24 (77.4%)	9 (29%)	2 (6.5%)	0 (0%)

**Table 2:** Drug use during the COVID-19 infectious phase (n=31)

Azithromycin	Chloroquine	Ivermectin	Corticosteroids
11 (35.5%)	3 (9.7%)	6 (19.4%)	9 (29%)

In participants, CRLTTI was present for a minimum length of time of two months, with a maximum duration of 24 months. The mean duration of symptoms was 10.8 months (SD 5.7). As regards specific taste perception, different tastes seemed to be affected differently by CRLTTI, with salt tending to have higher results compared to the average perception reduction of sweet, acidic, and bitter for hyper-concentrated solutions, as is demonstrated in Table 3. 

**Table 3:** Response to hyperconcentrated substances, with 10 being strong taste perception and 0 being ageusia.

	Sweet	Salty	Acid	Bitter
Mean	6.129	8.935	7.516	7.290
Median	7	10	8	8
Standard Deviation	3.344	1.931	2.528	3.002
IQR	5.000	1.500	3.000	4.500
Minimum	0	3	0	0
Maximum	10	10	10	10

Considering the sum of all tastes combined on a scale from 0-40, taste perception of hyper-concentrated solutions in all participants had a mean of 29.35 (SD 7.59) out of 40 points. 

Dysgeusia occurred in 38.7% of participants (n=12), with some participants reporting more than one altered taste, while in others, it was related to a single substance. Bitter taste distortion was the most prevalent in this sample and the only one with a statistical difference in expression compared to other tastes, followed by sweet and acid. Taste distortion distribution over different tastes can be observed in Table 4.

**Table 4:** Presence of taste distortion per taste evaluated, binomial test

Binomial Test					
	Level	Count	Total	Proportion	p
Sweet Taste Distortion	N	28	31	0.90	< 0.001
	Y	3	31	0.10	< 0.001
Salt Taste Distortion	N	31	31	1.00	< 0.001
Acid Taste Distortion	N	29	31	0.94	< 0.001
	Y	2	31	0.06	< 0.001
Bitter Taste Distortion	N	21	31	0.68	0.071
	Y	10	31	0.32	0.071
Note. Hₐ is proportion ≠ 0.5					

Regarding the vaccination status of the sample, a total of 25 participants (80.6%) developed taste impairment after COVID-19 infection (attested by PCR-RT test) without being vaccinated, while six (19.4%) developed taste impairment (attested by PCR-RT test) after full COVID-19 immunization. We did not have reports of CRLTTI development triggered by vaccination (one, two, or three doses). 

Furthermore, self-reported smell data is described in Table 5. One patient reported perfect smell recovery after a period with anosmia during the infectious phase; however, the taste impairment remained for a prolonged period. All other participants reported anosmia or parosmia associated with taste impairment. 

**Table 5:** Self-reported smell perception (0-10), with 0 complete anosmia and 10 smell perception before the COVID-19 infection.

	Self-Reported Olfact
N	31
Missing	0
Mean	3.84
Median	4
Standard deviation	2.48
Minimum	0
Maximum	10

Linear regression models were carried out to evaluate the influence of different factors on taste perception (0-40), taste distortion (0-40), and smell impairment (0-10), with results expressed in the tables below. No relationship was found between the factors and covariates evaluated when analyzing taste perception, taste distortion, and Olfact perception (Tables 6, 7). 

**Table 6:** Linear regression using taste perception (0-40) as dependent variable, regarding the relationship with other factors evaluated in this study and quantitative outcomes (age and diagnosis time) as covariates. *IP = COVID-19 Infectious Phase Continuous Medication = Any drug or vitamin used daily

Linear Regression				
Model Fit Measures				
Model	R	R²		
1	0.48	0.23		
Model Coefficients - Taste Response (0-40)				
Predictor	Estimate	SE	t	p
Intercept ᵃ	30.62	5.27	5.82	
Active Smoker:				
Y – N	7.34	6.56	1.12	0.276
Sex:				
F – M	1.21	3.85	0.32	0.756
Long COVID symptoms:				
Y – N	0.17	3.95	0.04	0.967
Previous Medical Conditions:				
Y – N	-1.52	4.13	-0.37	0.717
Continuous Medication Use:				
Y – N	-2.01	3.87	-0.52	0.610
Hypersensitivity:				
Y – N	1.04	3.67	0.28	0.779
Chloroquine:				
Y – N	-1.18	6.07	-0.19	0.848
Ivermectin:				
Y – N	-7.35	5.51	-1.34	0.197
Corticosteroids:				
Y – N	-1.51	4.36	-0.35	0.733
Azithromycin:				
Y – N	1.55	5.01	0.31	0.760
Age	0.05	0.15	0.31	0.762
Diagnosis Time (months)	0.06	0.32	0.18	0.856
ᵃ Represents reference level				
Assumption Checks				
Normality Test (Shapiro-Wilk)				
Statistic	p			
0.95	0.128			

**Table 7:** Linear regression using taste distortion (0-40) as dependent variable, regarding the relationship with other factors evaluated in this study and quantitative outcomes (age and diagnosis time) as covariates. *IP = COVID-19 Infectious Phase Continuous Medication = Any drug or vitamin used daily

Linear Regression				
Model Fit Measures				
Model	R	R²		
1	0.59	0.35		
Model Coefficients - Taste Distortion (0-40)				
Predictor	Estimate	SE	t	p
Intercept ᵃ	15.88	6.14	2.59	0.019
Active Smoker:				
Y – N	-2.37	6.00	-0.40	0.697
Sex:				
F – M	-0.83	3.42	-0.24	0.810
Long COVID symptoms:				
Y – N	-0.45	3.57	-0.13	0.900
Previous Medical Conditions:				
Y – N	-0.85	3.87	-0.22	0.828
Continuous Medication Use:				
Y – N	0.52	3.65	0.14	0.888
Hypersensitivity:				
Y – N	3.55	3.36	1.06	0.305
Chloroquine:				
Y – N	-7.50	5.35	-1.40	0.178
Ivermectin:				
Y – N	7.49	5.02	1.49	0.153
Corticosteroids:				
Y – N	0.12	3.87	0.03	0.975
Azithromycin:				
Y – N	-4.50	4.57	-0.98	0.339
Age	-0.22	0.12	-1.76	0.095
Diagnosis Time (months)	-0.14	0.27	-0.52	0.612
ᵃ Represents reference level				
Assumption Checks				
Normality Test (Shapiro-Wilk)				
Statistic	p			
0.98	0.852			

The analysis of Olfact perception (0-10) as an independent variable also did not show any relation with the same dependent variables evaluated in this study, as can be observed in Table 8.

**Table 8:** Linear regression using smell perception (0-10) as dependent variable, regarding the relationship with other factors evaluated in this study and quantitative outcomes (age and diagnosis time) as covariates. *IP = COVID-19 Infectious Phase Continuous Medication = Any drug or vitamin used daily

Linear Regression				
Model Fit Measures				
Model	R	R²		
1	0.46	0.21		
Model Coefficients - Olfact (0-10)				
Predictor	Estimate	SE	t	p
Intercept ᵃ	0.69	2.44	0.28	0.779
Active Smoker:				
Y – N	-0.01	2.38	-0.00	0.997
Sex:				
F – M	-0.29	1.36	-0.21	0.834
Long COVID symptoms:				
Y – N	0.99	1.42	0.70	0.494
Previous Medical Conditions:				
Y – N	-1.57	1.54	-1.02	0.321
Continuous Medication Use:				
Y – N	2.27	1.45	1.57	0.134
Hypersensitivity:				
Y – N	-0.22	1.33	-0.17	0.868
Chloroquine:				
Y – N	0.64	2.12	0.30	0.766
Ivermectin:				
Y – N	-1.34	1.99	-0.67	0.509
Corticosteroids:				
Y – N	0.75	1.54	0.49	0.629
Azithromycin:				
Y – N	0.46	1.82	0.25	0.802
Age	0.05	0.05	0.92	0.367
Diagnosis Time (months)	-0.02	0.11	-0.19	0.854
ᵃ Represents reference level				
Assumption Checks				
Normality Test (Shapiro-Wilk)				
Statistic	p			
0.95	0.168			

## Discussion

The average length of taste impairment found was 10.8 months (SD 5.7), with maximum symptoms lasting for a maximum of 24 months without recovery, which contradicted the initial evidence and perspectives shown in the literature based on transitory and rapid recovery after COVID-19 infection [2,8]. To the best of the authors’ knowledge, this is the first study to describe 24 months of taste and smell impairment in the Brazilian population. Viruses can damage structures and cause transitory anosmia and ageusia, with symptoms that are usually resolved within weeks. Whereas COVID-19 did not seem to follow this trend, causing the prolonged manifestation of symptoms when compared with SARS and other coronavirus types [9]. The causes and possible predictive factors related to the lack of taste and smell remain unknown, and we do not discard the possibility of permanent damage to gustatory and olfactory complexes caused by COVID-19 [10].

The mechanisms involved in alterations to acid, sweet, bitter, and salty taste perception due to COVID-19 contagion remain obscure, since we do not yet fully understand the etiopathology of COVID-19. The exact pathway by which COVID-19 alters taste perception and the explanation for the appearance of different symptoms between subjects remain unclear [11,12]. Therefore, as yet, there is no explanation for the possibility of certain tastes being more affected than others or for the presence of dysgeusia in either single or multiple tastes that can be observed in this study. One plausible explanation may be due to the composition of taste buds, which are composed of four basic cell types (I, II, III, IV), which are differently affected by COVID-19 invasion. Tissue invasion appears to be linked to type II cells of gustatory cells, which express angiotensin-converting enzyme 2 (ACE2), and the length of taste impairment occurs concomitantly with disruption of the epithelial stem cell layer [9,13,14].

The correlation between high serum IL-6 levels and taste impairment could also be another possible explanation for prolonged taste disturbances, in which the virus invasion may lead to chronic inflammatory conditions directly affecting the gustatory/olfactory systems. [15] The authors are aware of different possibilities that can lead to CRLTTI once both gustatory and olfactory systems combined guarantee correct flavor perception and changes in their physiology of the central nervous system (CNS) or peripheral nervous system (PNS) can lead to wrong taste/smell perception or even no perception at all [14]. 

Since ACE2 receptors expressed in the PNS are possibly also expressed in neuronal support cells associated with the gustatory and olfactory complex, this raises the possibility of direct damage caused to the PNS by COVID-19, which is another feasible explanation for the development and maintenance of CRLTTI [16]. In support of this finding, recent reports of neuropathic pain developed after COVID-19 contagion have been described, which were mainly caused by direct COVID-19 viral invasion of the PNS and consequently maintained through a persistent inflammatory state in this tissue [17].

The other possibility involved in the development of CRLTTI is a viral invasion of the CNS. The presence of symptoms of CNS invasion in the COVID-19 infected population has been well described, despite there being no consensus to explain the virus pathway for invasion and further consequences for the CNS [18]. When considering the development and perpetuation of CRLTTI, the presence of neurological alterations related to this condition is feasible. This is because malfunctioning or imbalance of homeostasis in all/any of these support structures and cells (CNS, PNS, olfactory, and gustatory complexes) might explain the development and perpetuation of these symptoms. The reasons why only a portion of infected people present with taste impairment and why some others demonstrate prolonged periods of taste impairment remain obscure [19-23].

Another interesting characteristic of this sample was the mean age of 40.26 years (SD 12.06), with women representing 71% (n=22) of the sample. These findings corroborate literature findings, which have demonstrated that young women not only have higher rates of taste impairment but are also followed by the expression of higher levels of long-lasting COVID-19 symptoms [9,24,25]. 

Participants diagnosed with previous chronic diseases and/or regular medication use were not factors that predicted taste impairment when using regression models. Moreover, medication use during the infectious phase did not cause taste impairment (Azithromycin, Corticosteroids, Chloroquine and Ivermectin), demonstrating that medication use during IP was not related to the development of CRLTTI. The great majority of the sample (80.6%) developed CRLTTI before the first dose of COVID-19 vaccine, despite the reports of fully immunized participants who also developed CRLTTI. This specific characteristic must be observed carefully and be the subject of future research.

It is noteworthy that our study only included participants with CRLTTI and that the test performed for assessment was developed by the researchers for this specific condition; thus, it lacks the validation of other studies for comparative analysis. We recommend that comparisons with the general population should be limited, and data must be carefully interpreted. Nevertheless, there are long-term COVID-19 effects among these prolonged symptoms (fatigue, shortness of breath, myalgia, articular pain, neuropathy, chest pain, dizziness/headache, and brain fog) that persist for two weeks or longer, characterized as “Long term COVID-19”. More specifically, the inclusion of taste and smell impairment within these conditions, considered “Long Term COVID-19,” may indicate that ageusia and dysgeusia could be related to the development of multiple conditions without being the most prevalent types [23]. Studies have estimated the manifestation of long-term COVID-19 symptoms in 10% of the infected population [26], and 23% of this population has shown prolonged taste disorders [23].

The presence of combined taste and smell impairments and their relationship has not yet been well established, as mentioned in the results found in this study, in which the majority of participants had concomitant taste and smell impairments. This was contrary to the study of Niklassen, who found no combined taste and smell impairment after the infectious phase of COVID-19 [26].

With regard to smell impairment, all participants had concomitant smell and taste loss in the infectious phase of COVID-19, with some individuals reporting higher levels of olfactory recovery after the infectious phase than that of their taste, or the opposite, in self-reported tests. Since this study was part of an RCT with experimental treatments targeted to the tongue, smell test evaluation was based on self-reported general routine perception, which could provide a general idea of how people were affected by COVID-19-related anosmia, limiting further conclusions. In contrast, this self-reported methodology is a specific but not sensitive method [27].

Limited sample size, restricted ethnic background, and also simplified taste evaluation and self-reported measurements can be considered as limitations of our study. Additionally, this study had participants with and without complete COVID-19 vaccination once this study started when the vaccine scheme was not fully available to all populations.

More studies are necessary to understand the causes and effects of COVID-19-related long-term taste impairment.

## Conclusions

The COVID-19 infection can trigger taste and smell disturbances that last as long as 24 months. Prolonged taste impairment seems to affect hyper-concentrated taste perception differently for different tastes. Medication use during the infectious phase, previous diseases, chronic medication use, tobacco use, and hypersensitivity to medication/food appear not to be linked to CRLTTI development. 

## Appendices

Questionnaire 

PATIENT NO:_________________________

Did you received any vaccine before the COVID-19 infection?

   🔲 YES     🔲 NO

If yes, please complete:   🔲 Complete Scheme (2 doses at least)    🔲 Incomplete Scheme (1 dose) 

Did you have any systemic condition/disease when diagnosed with COVID-19?    

              🔲 YES     🔲 NO

If yes, please specify the name. _______________________________________________________

Did you utilize any medication/vitamin on a daily basis when diagnosed with COVID-19?

              🔲 YES     🔲 NO

If yes, please specify the name and dosage. 

_______________________________________________________

Do you have any known allergies (food or medication)?  

                      🔲 YES     🔲 NO

If yes, please specify? ___________________________________________________

Time since COVID 19 diagnosis (months):_______________________

Do you have a PCR test attesting COVID-19 Infection?

                      🔲 YES    🔲 NO

Have you been hospitalized due COVID-19 infection? 

🔲 YES     🔲 NO

🔲 ICU  How long (days)?________

🔲 Oxygen use?  How long (days)?__________

🔲 Intubation? How long (days)?__________

Have you lost (completely or partially) your sense of taste for longer than 2 months?         

🔲 YES     🔲 NO

Have you lost (completely or partially) your sense of smell longer than 2 months? 

🔲 YES     🔲 NO

Additionally to taste impairment, do you remain with any of the symptoms listed below? 

🔲 tiredness/fatigue     🔲 shortness of breath    🔲 muscular pain (myalgia)    

🔲 joint pain  🔲 nerve pain      🔲chest pain    🔲headache    🔲memory problems

Have you used any of the medications listed below during the infectious phase of COVID 19? 

Chloroquine    🔲 YES     🔲 NO  Amount:______________

Ivermectin    🔲 YES     🔲 NO   Amount:______________

Azithromycin   🔲 YES     🔲 NO   Amount:______________

Corticosteroids     🔲 YES     🔲 NO Amount:______________

🔲 NONE OF THE ABOVE
